# Leg and arm adiposity is inversely associated with diastolic hypertension in young and middle-aged United States adults

**DOI:** 10.1186/s40885-021-00190-2

**Published:** 2022-01-15

**Authors:** Aayush Visaria, David Lo, Pranay Maniar, Bhoomi Dave, Parag Joshi

**Affiliations:** 1 Department of Medicine, Rutgers New Jersey Medical School, Newark, NJ USA; 2 North American Disease Intervention, Rutgers University, New Brunswick, NJ USA; 3 New Jersey Institute of Technology, Newark, NJ USA; 4 Division of Cardiology, Department of Medicine, University of Texas Southwestern Medical Center, Dallas, TX USA

**Keywords:** Body composition, Body fat distribution, Hypertension, Epidemiology, Blood pressure

## Abstract

**Background:**

We sought to determine the association between appendicular adiposity and hypertension, with the purpose of better understanding the role of body fat distribution on blood pressure (BP).

**Methods:**

We included 7411 adults aged 20 to 59 who were not taking antihypertensives and without cardiovascular disease from the 2011 to 2018 National Health and Nutrition Examination Surveys. Leg & arm adiposity, determined via dual-energy X-ray absorptiometry scans, was defined as percent of total body fat present in legs/arms (leg/total%, arm/total%). Measures were categorized into sex-specific tertiles. We estimated change in BP and odds ratios (ORs) of hypertension (BP ≥ 130/80) and hypertension subtypes using multivariable, survey design-adjusted linear & logistic regression, respectively.

**Results:**

Of the participants, 49% were female, the average (standard deviation) age was 37.4 (0.3) years, and 24% had hypertension. Those in the highest tertile (T3) of leg/total% had 30% decreased adjusted ORs (aOR) of hypertension compared to the lowest tertile (T1; aOR, 0.70; 95% confidence interval [95% CI], 0.55–0.89). This association was not significant for arm/total% (0.89, 0.68–1.17). T3 of leg/total% was associated with 49% lower, 41% lower, and unchanged relative odds of isolated diastolic hypertension (IDH), systolic-diastolic hypertension (SDH), and isolated systolic hypertension (ISH) compared to T1 (IDH: 0.51, 0.37–0.70; SDH: 0.59, 0.43–0.80; ISH: 1.06, 0.70–1.59). For every 10% increase in leg/total%, diastolic BP decreased by an adjusted mean 3.5 mmHg (95% CI, − 4.8 to − 2.2) in males and 1.8 mmHg (95% CI, − 2.8 to − 0.8) in females (*P* < 0.001 for both).

**Conclusions:**

A greater proportional distribution of fat around the legs is inversely, independently associated with hypertension, and more specifically, diastolic hypertension (IDH and SDH).

**Supplementary Information:**

The online version contains supplementary material available at 10.1186/s40885-021-00190-2.

## Background

Hypertension is a common chronic illness in the United States, with prevalence estimates greater than 100 million patients [1, 2]. There are several well-established risk factors for hypertension, including obesity, physical inactivity, high salt diet, and stress [2–6]. In addition, studies have demonstrated that hypertension subtypes (isolated diastolic hypertension, IDH; systolic-diastolic hypertension, SDH; isolated systolic hypertension, ISH) have unique age and demographic distributions and differences in significance of traditional risk factors [7, 8]. For example, elevated body mass index (BMI) was more significantly associated with IDH than either ISH or SDH in US adults [9].

While obesity has been associated with hypertension and downstream cardiovascular complications through mechanisms such as activation of the renin-angiotensin-aldosterone axis, increased cardiac output, sympathetic activation, insulin resistance, and adipocyte-related hormone release [10, 11], recent literature has provided evidence that distribution of body fat is just as, if not more, important than total body adiposity. Total body adiposity can be divided into appendicular adiposity (arm & leg adiposity), truncal adiposity, and head & neck adiposity. Truncal, visceral adiposity, often estimated by waist circumference, has been consistently positively associated with cardiometabolic conditions [11, 12]. Leg adiposity, on the other hand, has been inversely associated with cardiometabolic factors such as insulin resistance [13–17], metabolic syndrome [18–20], and cardiovascular disease & mortality [21–26] in several cross-sectional studies, albeit of questionable clinical significance. Additionally, the majority of leg adiposity is subcutaneous fat, which has been shown to be metabolically distinct to visceral adiposity. Subcutaneous fat is less likely to release sympathetic activating or renin-angiotensin-aldosterone axis activating substances and is also less likely to promote insulin resistance, suggesting a potential physiological mechanism for lower blood pressure (BP) [27–30].

Despite these studies, few studies have assessed BP individually [18, 19, 25, 31, 32] and thus, it remains unclear whether leg adiposity is independently associated with BP and hypertension. It is also unknown whether favorable associations for leg adiposity can be generalized to overall appendicular adiposity (both upper and lower extremities). There is a lack of recent, nationally representative studies assessing the role of appendicular adiposity in hypertension according to newer American College of Cardiology/American Heart Association (ACC/AHA) guidelines, which have markedly increased those classified as hypertensive. Furthermore, to our knowledge there exist no studies on the association between appendicular adiposity and hypertension subtype, which is important to deconstruct as studies have demonstrated elevated BMI as a primary risk factor for IDH in young adults [9, 18–20].

Hence, in this study, we investigate the association between various appendicular adiposity measures and BP, hypertension, and hypertension subtypes in young- to middle-aged US adults, with the purpose of better understanding the role of body fat distribution and its potential for early identification of hypertension and cardiovascular risk.

## Methods

### National health and nutrition examination survey dataset and linkage

The National Health and Nutrition Examination Survey (NHANES) is a nationally representative survey of the civilian, noninstitutionalized US population. The survey, implemented by the National Center for Health Statistics, follows a complex, stratified, multistage probability design. Surveys are conducted in 2-year cycles. We combined four publicly available cycles (2011–2012, 2013–2014, 2015–2016, and 2017–2018 cycles) to create a final NHANES 2011 to 2018 dataset for this secondary analysis. Further information about the dataset and the sample design can be found elsewhere [33].

### Study population

Among the total 39,426 participants included in the NHANES 2011 to 2018 surveys, 11,222 were aged 20 to 59 and had dual-energy X-ray absorptiometry (DXA) scan data (*N* = 28,204 excluded). Among these participants, a further 745 were excluded due to self-reported history of cardiovascular disease (stroke, myocardial infarction, coronary artery disease, and/or heart failure). An additional 1706 who were taking antihypertensive medications and 1360 with missing data on covariates were excluded, yielding the final sample size of 7411 (3723 male and 3688 non-pregnant female) (Fig. S1).

### Variables of interest

Our primary predictor was the average percentage of whole-body fat mass present in the lower extremities (termed leg/total%) and upper extremities (arm/total%). Secondary predictors included (1) the average lean mass-to-fat mass ratio between each lower extremity (termed leg lean/fat ratio) or upper extremity (arm lean/fat ratio), and (2) average percentage of extremity mass (excluding bone mineral content) that was fat (leg fat%, arm fat%). Trained and certified radiology technicians acquired and calculated these measurements through a whole-body scan using Hologic Discovery model A densitometers (Hologic Inc., Bedford, MA, USA). Further details about the DXA scan and quality control measures are available elsewhere [34]. Each predictor was also categorized into sex-specific tertiles—leg/total% (female: < 36, 36–41%, ≥41%; male: < 31.5, 31.5–36.0%, ≥36%); leg lean/fat ratio (female: < 1.1, 1.1–1.4, ≥1.4; male: < 2.3, 2.3–3.0, ≥3.0); leg fat % (female: < 40, 40–45%, ≥45%; male: < 24, 24–29%, ≥29%); arm/total % (female: < 11.6, 11.6–12.9%, ≥12.9%; male: < 11.3, 11.3–12.2%, ≥12.2%); arm lean/fat ratio (female: < 1.1, 1.1–1.5, ≥1.5; male: < 2.6, 2.6–3.5, ≥3.5); arm fat % (female: < 39, 39–45%, ≥45% male: < 21, 21–26%, ≥26%).

Our primary outcomes included hypertension and hypertension subtype. Hypertension was diagnosed as per the 2017 ACC/AHA guidelines (≥130/80 mmHg) [35] and hypertension subtypes were diagnosed as follows: (1) ISH: systolic BP (SBP) ≥130 and diastolic BP (DBP) < 80; (2) IDH: SBP < 130 and DBP ≥80; (3) SDH: SBP ≥130 and DBP ≥80; (4) normotension: SBP < 130 and DBP < 80.

Secondary outcomes included SBP, DBP, and pulse pressure, treated as continuous variables. Auscultatory, sitting BP measurements were performed using a mercury sphygmomanometer (Baumanometer® - Gravity Rx model; W.A. Baum Co., Inc., Copiague, NY, USA). All staff followed strict quality control and protocol-driven procedures that are available elsewhere [36]. BP was measured three times consecutively by a trained physician during a mobile examination center visit. Participants were asked to sit and rest for 5 min before the first measurement. There was a thirty-second interval between measurements. We took the average of the three BP measurements.

Our covariates included demographic factors (age, race/ethnicity, poverty-income ratio [median household income/poverty threshold]), sociobehavioral factors (education level, physical activity, alcohol consumption, smoking status as determined by serum cotinine levels), and cardiometabolic variables (BMI, waist circumference, high density lipoprotein [HDL], triglycerides, C-reactive protein, diabetes status [hemoglobin A1c, HbA1c > 6.5%, fasting glucose ≥126 mg/dL, self-reported diagnosis of diabetes, oral or insulin medication use], serum creatinine, and alanine aminotransferase). Measurement protocols for each covariate are described elsewhere [36].

### Statistical analysis

As an initial, exploratory analysis, we first plotted a 20% representative subsample of the data and determined the correlation between BP and leg adiposity measures, stratified by sex. We then used multivariable linear regression, assuming normality, to estimate mean changes in BP overall and stratified by BMI group. As sex did not significantly modify the effect of leg adiposity on BP (P for interaction = 0.25), subsequent analyses used sex-specific categories, but did not stratify by sex.

To compare baseline characteristics across leg/total% tertiles, we used one-way ANOVA for continuous variables and Rao-Scott chi-square tests for categorical variables. We also compared baseline characteristics across hypertension status in both males and females and used identical statistical analyses. To determine the independent association between appendicular adiposity and overall hypertension, we used multivariable logistic regression, adjusting for covariates in a series of three additive models. Model 1 adjusted for demographic factors (age, sex, race/ethnicity, poverty index). Model 2 adjusted for Model 1 factors + sociobehavioral and cardiometabolic factors (SBP, heart rate, triglycerides, BMI, truncal fat mass, HDL, alanine aminotransferase, diabetes status, serum creatinine, albuminuria, smoking, alcohol consumption). Model 3 adjusts for other examination factors (leg fat parameters other than main predictor, arm fat parameters other than main predictor). There was no significant multicollinearity between covariates as determined by variance inflation factor. To determine the independent association between predictors and hypertension subtypes, we estimated relative risk ratios (RRRs) using multinomial logistic regression in a series of three additive models as described for the logistic regression. Finally, we determined the predictive value of our adiposity measures using the c-statistic, an estimate of the area-under-receiver operator curve (AUC).

Analysis was stratified by BMI group (low/normal, overweight, and obese) to account for differences in fat distribution and content. All analyses utilized weights to produce nationally representative estimates and account for the oversampling of minorities (e.g., elderly, African Americans, and Mexican Americans). Specifically, we utilized NHANES examination sample weights for each cycle and divided by 4 to obtain pooled weights across four survey cycles. We did not adjust for statistical significance for multiple comparisons. All analyses also accounted for the complex survey design with strata and cluster variables using a traditional Taylor linearization approach to obtain accurate variance estimates using SAS survey procedures. All analyses followed NHANES analytic guidelines [33]. Analyses were conducted using SAS ver. 9.4 (SAS Institute Inc., Cary, NC, USA) with a significance level of 0.05.

## Results

Among the 7411 participants, the average age (standard deviation, SD) of the 3723 males and 3688 females was 36.9 (0.3) and 37.8 (0.3) years, respectively. Males who had hypertensive BPs (1158, 29.7%) were older, had higher BMI, truncal fat, and waist circumference, higher triglyceride levels, and higher HbA1c%. On the contrary, hypertensive males had lower leg/total% and leg and arm lean/fat ratio, but no difference in arm/total%, HDL, and low-density lipoprotein (LDL) compared to normotensive males (Table S1). Females followed similar patterns in baseline characteristics (Table S2).

When comparing baseline characteristics by sex-specific tertiles of leg/total%, those in the highest tertile (T3) of leg/total% were younger, less educated, and of lower BMI and waist circumference. They also had lower leg lean/fat ratio, larger average leg area and upper leg length, higher arm lean/fat ratio, and no significant difference in average arm area. BP was significantly lower with increasing leg/total% tertile. Further, those in T3 had a more favorable metabolic and lipid panel, with a 38.2% difference in prevalence of hypertriglyceridemia and 26% difference in impaired glucose tolerance between the lowest and highest tertiles (Table 1).

**Table 1:** Baseline characteristics by leg adiposity (leg/total%) tertile

Variable	Tertile 1^a^	Tertile 2^a^	Tertile 3^a^	*P*-value^b^
Demographics				
Age (yr)	41.6 (0.3)	37.5 (0.3)	33.7 (0.4)	< 0.0001
Race/ethnicity				
Mexican American	672 (19.6%)	357 (10.9%)	171 (5.0%)	< 0.0001
Other Hispanic	295 (8.7%)	274 (7.8%)	219 (6.3%)	
Non-Hispanic White	734 (56.5%)	857 (61.3%)	1037 (66.6%)	
Non-Hispanic Black	199 (4.6%)	395 (8.5%)	735 (14.4%)	
Non-Hispanic Asian	417 (7.2%)	450 (7.5%)	301 (4.6%)	
Other race	87 (3.3%)	119 (4.0%)	92 (3.1%)	
Poverty-income ratio	2.9 (0.06)	3.0 (0.06)	3.0 (0.08)	0.1095
Education level				
< High school	1300 (62.1%)	1552 (66.9%)	1706 (71.8%)	< 0.0001
High school	514 (21.2%)	519 (21.3%)	540 (19.5%)	
> High school	590 (16.7%)	381 (11.8%)	309 (8.7%)	
Sociobehavioral factors				
Smoking status^c^				
Low/none	829 (37.4%)	746 (33.6%)	672 (32.4%)	0.0070
Moderate	972 (36.4%)	1047 (40.5%)	1032 (37.7%)	
High	603 (26.2%)	659 (26.0%)	847 (29.9%)	
Alcohol consumption (drinks/day)	3.3 (0.3)	3.2 (0.3)	3.1 (0.2)	0.7734
Examination measurements				
BMI (kg/m^2^)	30.6 (0.2)	28.0 (0.2)	25.5 (0.2)	< 0.0001
Waist circumference (cm)	103.4 (0.4)	95.8 (0.4)	87.7 (0.4)	< 0.0001
Truncal fat percent (%)	34.9 (0.2)	31.6 (0.2)	26.9 (0.2)	< 0.0001
% Total fat in lower extremities^d^	30.3 (0.1)	36.1 (0.1)	42.2 (0.1)	< 0.0001
Leg lean mass/fat mass ratio^d^	2.1 (0.03)	2.0 (0.03)	1.9 (0.02)	< 0.0001
% Fat in total leg mass^d^	33.4 (0.3)	35.3 (0.3)	35.9 (0.3)	< 0.0001
Average leg area (cm^2^)	365.8 (1.4)	371.7 (1.2)	376.9 (1.3)	< 0.0001
Upper leg length (cm)	38.7 (0.1)	39.9 (0.1)	40.6 (0.1)	< 0.0001
% Total fat in upper extremities	12.4 (0.04)	12.0 (0.04)	11.7 (0.04)	< 0.0001
Arm lean mass/fat mass ratio	2.1 (0.03)	2.3 (0.03)	2.5 (0.03)	< 0.0001
% Fat in total arm mass	35.0 (0.3)	33.3 (0.3)	30.8 (0.3)	< 0.0001
Average arm area (cm^2^)	233.0 (1.1)	232.2 (0.9)	231.4 (0.9)	0.5127
Heart rate (bpm)	73.7 (0.4)	73.1 (0.3)	71.6 (0.3)	< 0.0001
SBP (mmHg)	120.3 (0.4)	116.0 (0.3)	113.7 (0.3)	< 0.0001
DBP (mmHg)	73.7 (0.3)	70.7 (0.3)	68.1 (0.3)	< 0.0001
Pulse pressure (mmHg)	46.6 (0.4)	45.3 (0.4)	45.6 (0.4)	0.031
Average maximum SBP (mmHg)	123.2 (0.4)	118.8 (0.3)	116.5 (0.3)	< 0.0001
Maximum – minimum SBP (mmHg)	5.8 (0.1)	5.6 (0.1)	5.6 (0.1)	0.18
Maximum – minimum DBP (mmHg)	5.7 (0.1)	5.9 (0.2)	6.1 (0.2)	0.027
Laboratory measurements				
Plasma glucose (mg/dL)	109.2 (0.9)	97.6 (0.6)	94.8 (0.5)	< 0.0001
Hemoglobin A1c (%)	5.7 (0.03)	5.4 (0.01)	5.2 (0.01)	< 0.0001
Serum insulin (uU/mL)	16.1 (0.8)	10.5 (0.4)	8.0 (0.3)	< 0.0001
Serum HDL (mg/dL)	47.7 (0.4)	53.6 (0.5)	58.8 (0.5)	< 0.0001
Serum LDL (mg/dL)	123.6 (2.5)	116.3 (1.8)	106.0 (1.7)	< 0.0001
Serum triglycerides (mg/dL)	197.0 (4.0)	134.3 (2.5)	98.6 (1.4)	< 0.0001
ALT (IU/L)	29.9 (0.5)	24.9 (0.5)	20.9 (0.3)	< 0.0001
AST (IU/L)	26.1 (0.5)	24.2 (0.3)	23.0 (0.2)	< 0.0001
Serum albumin (g/dL)	4.29 (0.01)	4.33 (0.01)	4.39 (0.01)	< 0.0001
Serum creatinine	0.83 (0.01)	0.85 (0.01)	0.85 (0.01)	0.0006
Serum creatine phosphokinase	152.2 (5.0)	159.1 (6.8)	157.5 (5.2)	0.6855
Comorbidities				
Impaired glucose tolerance^e^	1138 (43.3%)	751 (24.6%)	516 (16.9%)	< 0.0001
Hypertriglyceridemia	1258 (53.1%)	753 (30.5%)	381 (14.9%)	< 0.0001
Anti-diabetes medication use	148 (4.5%)	32 (0.8%)	10 (0.3%)	< 0.0001
Cholesterol medication use	161 (7.7%)	74 (3.6%)	29 (1.6%)	< 0.0001
HOMA-IR^f^	474 (54.5%)	271 (27.5%)	144 (13.8%)	< 0.0001

Continuous variables represented as weighted mean (SD). Categorical variables represented as unweighted N (weighted %)

*SD* standard deviation, *BMI* body mass index, *SBP* systolic blood pressure, *DBP* diastolic blood pressure, *HDL* high density lipoprotein, *LDL* low-density lipoprotein, *ALT* alanine aminotransferase, *AST* aspartate aminotransferase, *HOMA-IR* Homeostatic Model Assessment of Insulin Resistance

^a^Tertiles are of leg/total % (percentage of total body fat mass that is in bilateral lower extremities). Sex-specific tertiles are as follows: female: < 36, 36–41%, ≥41%; male: < 31.5, 31.5–36.0%, ≥36%

^b^*P*-value calculated using one-way ANOVA for continuous variables and Rao-Scott chi-square tests for categorical variables. *P*-values and/or significance levels were not adjusted for multiple comparisons as this table was considered exploratory in nature

^c^Smoking categories determined by serum cotinine levels

^d^% Total fat in lower extremities corresponds to leg/total %, leg lean mass/fat mass ratio corresponds to leg lean/fat ratio, % fat in total leg mass corresponds to leg fat %. Identical for arm measures

^e^Impaired glucose tolerance was defined as hemoglobin A1c ≥5.7%, fasting plasma glucose ≥100 mg/dL, self-reported history of diabetes or self-reported history of oral anti-diabetes medications or insulin therapy

^f^Data on HOMA-IR only available for 2756 individuals (614 with hypertension). HOMA = fasting glucose (mg/dL) × insulin (uU/mL) / 405. HOMA ≥3 signifies insulin resistance

Scatterplots of leg adiposity measures with SBP and DBP showed significant negative correlations for leg lean/fat ratio and leg/total%, but a positive correlation for leg fat%. Leg/total% showed the greatest magnitude of correlation, at r = 0.20 in males and r = 0.25 in females (Fig. S2). Sex did not significantly modify the association between leg adiposity and BP (P for interaction = 0.25).

Nearly 24% of participants had hypertension. Those in T3 of leg/total% had 30% lower adjusted ORs (aORs) of hypertension compared to the lowest tertile (T1; aOR, 0.70; 95% confidence interval [CI], 0.55–0.89) (Table 2). Those in tertile 2 also displayed significantly reduced odds of hypertension compared to T1 (aOR, 0.81; 95% CI, 0.66–0.99). These associations did not stay significant for leg lean/fat ratio and leg fat% tertiles, losing their significance in Model 2, after adjustment for cardiometabolic factors, most importantly SBP. The corresponding tertiles for arm measures did not yield significant associations with hypertension (Table 3).

**Table 2:** Odds of hypertension by tertiles of leg adiposity measures

Variable		Tertile 1	Tertile 2	Tertile 3
No. (%)	1853 (23.8)	–	–	–
Leg/total %	Unadjusted	1.00 (Ref)	0.57 (0.47, 0.69)	0.35 (0.29, 0.42)
	Model 1	1.00 (Ref)	0.63 (0.52, 0.78)	0.44 (0.36, 0.54)
	Model 2	1.00 (Ref)	0.79 (0.65, 0.97)	0.68 (0.56, 0.82)
	Model 3	1.00 (Ref)	0.81 (0.66, 0.99)	0.70 (0.55, 0.89)
Leg lean/fat ratio	Unadjusted	1.00 (Ref)	0.83 (0.69, 0.98)	0.69 (0.57, 0.83)
	Model 1	1.00 (Ref)	0.86 (0.73, 1.02)	0.74 (0.61, 0.90)
	Model 2	1.00 (Ref)	1.19 (0.99, 1.43)	1.37 (1.07, 1.74)
	Model 3	1.00 (Ref)	1.11 (0.92, 1.34)	1.23 (0.95, 1.60)
Leg fat %	Unadjusted	1.00 (Ref)	1.24 (1.01, 1.53)	1.44 (1.19, 1.73)
	Model 1	1.00 (Ref)	1.20 (0.97, 1.45)	1.38 (1.14, 1.70)
	Model 2	1.00 (Ref)	0.90 (0.74, 1.11)	0.75 (0.59, 0.95)
	Model 3	1.00 (Ref)	0.93 (0.75, 1.15)	0.82 (0.63, 1.05)

Data are presented as odds ratio (95% confidence interval)

Model 1 adjusts for demographic factors (age, sex, race/ethnicity, poverty index). Model 2 adjusts for Model 1 factors + cardiometabolic factors (systolic blood pressure, heart rate, triglycerides, body mass index, truncal fat mass, high density lipoprotein, alanine aminotransferase, diabetes status, serum creatinine, albuminuria, smoking, alcohol consumption). Model 3 adjusts for other examination factors (leg fat parameters other than main predictor, arm fat parameters other than main predictor). Lean/fat ratio and fat % variables were not included in the same model due to significant correlation and high variance inflation factor. % Total fat in lower extremities corresponds to leg/total %, leg lean mass/fat mass ratio corresponds to leg lean/fat ratio, % fat in total leg mass corresponds to leg fat %. Identical for arm measures. Tertiles are as follows: leg/total % (female: < 36, 36–41%, ≥41%; male: < 31.5, 31.5–36.0%, ≥36%); leg lean/fat ratio (female: < 1.1, 1.1–1.4, ≥1.40; male: < 2.3, 2.3–3.0, ≥3.0); leg fat % (female: < 40, 40–45%, ≥45%; male: < 24, 24–29%, ≥29%)

**Table 3:** Odds of hypertension by tertiles of arm adiposity measures

Variable		Tertile 1	Tertile 2	Tertile 3
No. (%)	1853 (23.8)	–	–	–
Arm/total %	Unadjusted	1.00 (Ref)	0.97 (0.79, 1.18)	1.12 (0.92, 1.36)
	Model 1	1.00 (Ref)	0.99 (0.72, 1.35)	1.24 (0.87, 1.75)
	Model 2	1.00 (Ref)	0.96 (0.70, 1.31)	0.90 (0.64, 1.24)
	Model 3	1.00 (Ref)	0.87 (0.62, 1.22)	0.89 (0.68, 1.17)
Arm lean/fat ratio	Unadjusted	1.00 (Ref)	0.74 (0.64, 0.87)	0.50 (0.42, 0.60)
	Model 1	1.00 (Ref)	0.77 (0.42, 1.40)	0.63 (0.36, 1.11)
	Model 2	1.00 (Ref)	0.99 (0.73, 1.33)	1.17 (0.81, 1.70)
	Model 3	1.00 (Ref)	1.03 (0.81, 1.31)	1.13 (0.71, 1.80)
Arm fat %	Unadjusted	1.00 (Ref)	1.49 (1.26, 1.77)	2.03 (1.72, 2.40)
	Model 1	1.00 (Ref)	1.26 (0.95, 1.69)	1.66 (1.02, 2.69)
	Model 2	1.00 (Ref)	0.84 (0.65, 1.10)	0.92 (0.67, 1.25)
	Model 3	1.00 (Ref)	1.07 (0.63, 1.80)	0.97 (0.58,1.61)

Data are presented as odds ratio (95% confidence interval)

Model 1 adjusts for demographic factors (age, sex, race/ethnicity, poverty index). Model 2 adjusts for Model 1 factors + cardiometabolic factors (systolic blood pressure, heart rate, triglycerides, body mass index, truncal fat mass, high density lipoprotein, alanine aminotransferase, diabetes status, serum creatinine, albuminuria, smoking, alcohol consumption). Model 3 adjusts for other examination factors (leg fat parameters other than main predictor, arm fat parameters other than main predictor)

% Total fat in upper extremities corresponds to arm/total %, arm lean mass/fat mass ratio corresponds to arm lean/fat ratio, % fat in total arm mass corresponds to arm fat %. Tertiles are as follows: arm/total % (female: < 11.6, 11.6–12.9%, ≥12.9%; male: < 11.3, 11.3–12.2%, ≥12.2%); arm lean/fat ratio (female: < 1.1, 1.1–1.5, ≥1.5; male: < 2.6, 2.6–3.5, ≥3.5); arm fat % (female: < 39, 39–45%, ≥45%; male: < 21, 21–26%, ≥26%)

Stratifying by BMI group revealed attenuated, lower odds of hypertension for leg/total% with increasing BMI, although differences between BMI groups remained insignificant (P for interaction = 0.40) (Table 4). Among the low/normal BMI group, those in T3 of leg/total% had 46% lower adjusted odds of hypertension compared to T1. Among overweight individuals and obese individuals, there was no significant difference in odds of hypertension between T3 and T1 (Table 4). Overweight individuals in T3 of leg lean/fat ratio and arm lean/fat ratio had 73 and 102%, respectively, higher odds of hypertension compared to T1.

**Table 4:** Association between appendicular adiposity and HTN by BMI group

Variable		BMI group		
		Low/normal	Overweight	Obese
No. (% HTN)		443 (16.9)	612 (31.7)	798 (52.1)
Leg/total %	Unadjusted	0.30 (0.21, 0.43)	0.40 (0.28, 0.58)	0.79 (0.58, 1.07)
	Fully adjusted^a^	0.53 (0.30, 0.96)	0.91 (0.58, 1.43)	0.92 (0.63, 1.33)
Arm/total %	Unadjusted	1.24 (0.87, 1.75)	0.90 (0.64, 1.24)	0.89 (0.68, 1.17)
	Fully adjusted	1.00 (0.69, 1.45)	0.87 (0.62, 1.23)	0.85 (0.62, 1.16)
Leg lean/fat ratio	Unadjusted	1.01 (0.63, 1.61)	1.45 (1.04, 2.02)	1.05 (0.72, 1.53)
	Fully adjusted	1.00 (0.54, 1.84)	1.73 (1.16, 2.56)	1.20 (0.75, 1.91)
Arm lean/fat ratio	Unadjusted	0.63 (0.36, 1.11)	1.17 (0.81, 1.70)	1.13 (0.71, 1.80)
	Fully adjusted	0.92 (0.48, 1.77)	2.02 (1.29, 3.17)	1.43 (0.93, 2.20)
Leg fat%	Unadjusted	1.15 (0.78, 1.69)	0.61 (0.44, 0.86)	1.02 (0.69, 1.49)
	Fully adjusted	1.20 (0.63, 2.29)	0.63 (0.38, 1.03)	1.15 (0.66, 1.98)
Arm fat%	Unadjusted	1.66 (1.02, 2.69)	0.92 (0.67, 1.25)	0.97 (0.58, 1.61)
	Fully adjusted	1.08 (0.54, 2.14)	0.74 (0.44, 1.26)	0.76 (0.45, 1.30)

Data are presented as odds ratio (95% confidence interval). Odds ratio represent highest tertile vs. lowest tertile for each adiposity measure

*HTN* hypertension, *BMI* body mass index, *OR* odds ratio

^a^Fully adjusted model is identical to Model 3. Model 3 adjusts for other examination factors (leg fat parameters other than main predictor, arm fat parameters other than main predictor). Lean/fat ratio and fat % variables were not included in the same model due to significant correlation and high variance inflation factor

% Total fat in lower extremities corresponds to leg/total %, leg lean mass/fat mass ratio corresponds to leg lean/fat ratio, % fat in total leg mass corresponds to leg fat %. Identical for arm measures. Tertiles are as follows: leg/total % (female: < 36, 36–41%, ≥41%; male: < 31.5, 31.5–36.0%, ≥36%); leg lean/fat ratio (female: < 1.10, 1.10–1.40, ≥1.40; male: < 2.30, 2.30–3.00, ≥3.00); leg fat % (female: < 40, 40–45%, ≥45%; male: < 24, 24–29%, ≥29%). Arm measures: arm/total % (female: < 11.6, 11.6–12.9%, ≥12.9%; male: < 11.3, 11.3–12.2%, ≥12.2%); arm lean/fat ratio (female: < 1.1, 1.1–1.5, ≥1.5; male: < 2.6, 2.6–3.5, ≥3.5); arm fat % (female: < 39, 39–45%, ≥45%; male: < 21, 21–26%, ≥26%)

To investigate the association between leg/total% and hypertension, we divided hypertension into its subtypes and determined the RRR of each hypertension subtype relative to normotension (Table 5, Fig. S3). Those in T3 of leg/total% had 49% decreased odds of IDH, 41% decreased odds of SDH, but no significant change in odds of ISH. Arm adiposity measures followed similar patterns, although were of lesser significance. Unique to arm adiposity measures was a significant 46% decrease in the relative odds of ISH for T3 of arm fat% as well as a 111% increase in the relative odds of ISH for T3 of arm lean/fat ratio compared to T1 (Table 5).

**Table 5:** Association between leg adiposity and hypertension subtypes

Variable		Tertile 1	Tertile 2	Tertile 3
Leg/total%	Normotension	1.00 (Ref)		
	IDH	1.00 (Ref)	0.79 (0.59, 1.06)	0.51 (0.37, 0.70)
	SDH	1.00 (Ref)	0.70 (0.50, 0.98)	0.59 (0.43, 0.80)
	ISH	1.00 (Ref)	0.84 (0.61, 1.16)	1.06 (0.70, 1.59)
Leg lean/fat ratio	Normotension	1.00 (Ref)		
	IDH	1.00 (Ref)	0.99 (0.73, 1.34)	0.93 (0.62, 1.38)
	SDH	1.00 (Ref)	1.26 (0.95, 1.67)	1.50 (0.97, 2.32)
	ISH	1.00 (Ref)	1.16 (0.83, 1.63)	1.56 (1.00, 2.41)
Leg fat %	Normotension	1.00 (Ref)		
	IDH	1.00 (Ref)	1.17 (0.89, 1.54)	1.04 (0.72, 1.52)
	SDH	1.00 (Ref)	0.79 (0.55, 1.13)	0.67 (0.44, 1.04)
	ISH	1.00 (Ref)	0.76 (0.54, 1.08)	0.69 (0.43, 1.10)
Arm/total%	Normotension	1.00 (Ref)		
	IDH	1.00 (Ref)	0.99 (0.73, 1.34)	0.70 (0.52, 0.95)
	SDH	1.00 (Ref)	0.85 (0.62, 1.16)	0.75 (0.55, 1.02)
	ISH	1.00 (Ref)	1.16 (0.82, 1.64)	1.21 (0.88, 1.65)
Arm lean/fat ratio	Normotension	1.00 (Ref)		
	IDH	1.00 (Ref)	1.11 (0.82, 1.50)	1.12 (0.76, 1.65)
	SDH	1.00 (Ref)	1.13 (0.82, 1.54)	1.30 (0.84, 2.01)
	ISH	1.00 (Ref)	1.28 (0.94, 1.75)	2.11 (1.28, 3.46)
Arm fat%	Normotension	1.00 (Ref)		
	IDH	1.00 (Ref)	0.94 (0.71, 1.25)	0.96 (0.66, 1.41)
	SDH	1.00 (Ref)	0.93 (0.64, 1.33)	0.72 (0.46, 1.14)
	ISH	1.00 (Ref)	0.64 (0.45, 0.92)	0.54 (0.34, 0.86)

Data are presented as relative risk ratio (95% confidence interval)

*IDH* isolated diastolic hypertension, *SDH* systolic-diastolic hypertension, *ISH* isolated systolic hypertension

All estimates shown are fully adjusted via Model 3. Model 3 adjusts for other examination factors (leg fat parameters other than main predictor, arm fat parameters other than main predictor). Lean/fat ratio and fat % variables were not included in the same model due to significant correlation and high variance inflation factor

% Total fat in lower extremities corresponds to leg/total %, leg lean mass/fat mass ratio corresponds to leg lean/fat ratio, % fat in total leg mass corresponds to leg fat %. Identical for arm measures. Tertiles are as follows: leg/total % (female: < 36, 36–41%, ≥41%; male: < 31.5, 31.5–36.0%, ≥36%); leg lean/fat ratio (female: < 1.10, 1.10–1.40, ≥1.40; male: < 2.30, 2.30–3.00, ≥3.00); leg fat % (female: < 40, 40–45%, ≥45%; male: < 24, 24–29%, ≥29%). % Total fat in upper extremities corresponds to arm/total %, arm lean mass/fat mass ratio corresponds to arm lean/fat ratio, % fat in total arm mass corresponds to arm fat %. Tertiles are as follows: arm/total % (female: < 11.6, 11.6–12.9%, ≥12.9%; male: < 11.3, 11.3–12.2%, ≥12.2%); arm lean/fat ratio (female: < 1.1, 1.1–1.5, ≥1.5; male: < 2.6, 2.6–3.5, ≥3.5); arm fat % (female: < 39, 39–45%, ≥45%; male: < 21, 21–26%, ≥26%)

As an exploratory analysis, we treated appendicular adiposity and BP as continuous and did identical analyses stratified by sex (Tables 6, S3–S9). For every 1% increase in leg/total%, DBP decreased by an adjusted mean 0.35 mmHg (95% CI, − 0.48 to − 0.22) in males and 0.18 mmHg (95% CI, − 0.28 to − 0.08) in females (Table 6). This association did not hold for SBP in males. Leg lean/fat ratio was positively associated with SBP, while leg fat% was negatively associated with SBP but not DBP in both males and females (β [95% CI]; male: lean/fat ratio, 1.11 [0.34 to 1.89]; leg fat%, − 0.22 [− 0.34 to − 0.09]; female: lean/fat ratio, 2.15 [0.66 to 3.65]); leg fat%, − 0.16 [− 0.26 to − 0.06]). Arm adiposity measures demonstrated similar associations, with the exception that in males, arm fat% was inversely associated with DBP as well as SBP (Table S5). By comparison, every 1% increase in truncal fat results in an average DBP increase of 0.084 mmHg (95% CI, 0.003 to 0.165) and an SBP decrease of 0.148 mmHg (95% CI, 0.06 to 0.235).

**Table 6:** Association between leg adiposity and blood pressure

Variable			SBP		DBP		Pulse Pressure	
			β (95% CI)	*P*-value	β (95% CI)	*P*-value	β (95% CI)	*P*-value
No. (%)	1853 (23.8)		–	–	–	–	–	–
Leg/total%	Male	Unadjusted	− 0.48 (− 0.58, − 0.39)	< 0.0001	− 0.61 (− 0.70, − 0.53)	< 0.0001	0.13 (0.021, 0.24)	0.0199
		Fully adjusted	− 0.13 (− 0.29, 0.022)	0.093	− 0.35 (− 0.48, − 0.22)	< 0.0001	0.21 (0.042, 0.37)	0.015
	Female	Unadjusted	−0.53 (− 0.62, − 0.44)	< 0.0001	−0.31 (− 0.36, − 0.24)	< 0.0001	−0.23 (− 0.31, − 0.15)	< 0.0001
		Fully adjusted	− 0.17 (− 0.31, − 0.034)	0.015	−0.18 (− 0.28, − 0.08)	0.0007	−0.01 (− 0.15, 0.14)	0.94
Leg lean/fat ratio	Male	Unadjusted	−0.84 (−1.39, − 0.29)	0.0035	− 1.29 (− 1.73, − 0.86)	< 0.0001	0.46 (− 0.11, 1.03)	0.11
		Fully adjusted	1.11 (0.34, 1.89)	0.0058	− 0.42 (− 1.24, 0.40)	0.31	1.53 (0.54, 2.52)	0.0029
	Female	Unadjusted	−2.67 (−3.87, −1.47)	< 0.0001	−1.72 (− 2.76, − 0.69)	0.0015	−0.95 (− 2.31, 0.41)	0.17
		Fully adjusted	2.15 (0.66, 3.65)	0.0054	0.18 (−1.26, 1.62)	0.80	1.97 (0.25, 3.70)	0.025
Leg fat%	Male	Unadjusted	0.14 (0.05, 0.22)	0.0026	0.17 (0.11, 0.23)	< 0.0001	−0.032 (−0.11, 0.047)	0.43
		Fully adjusted	−0.22 (− 0.34, − 0.088)	0.0013	0.046 (− 0.084, 0.18)	0.48	−0.26 (− 0.42, − 0.11)	0.0011
	Female	Unadjusted	0.17 (0.10, 0.25)	< 0.0001	0.10 (0.036, 0.16)	0.0026	0.074 (−0.0094, 0.16)	0.081
		Fully adjusted	−0.16 (− 0.26, − 0.059)	0.0024	−0.032 (− 0.13, 0.061)	0.49	−0.13 (− 0.25, − 0.01)	0.036

*SBP* systolic blood pressure, *DBP* diastolic blood pressure, *β* regression parameter estimate, *CI* confidence interval (all estimates are of the fully adjusted model)

Fully adjusted model is identical to Model 3 and adjusts for demographic factors (age, sex, race/ethnicity, poverty index), cardiometabolic factors (SBP, heart rate, triglycerides, body mass index, truncal fat mass, high density lipoprotein, alanine aminotransferase, diabetes status, serum creatinine, albuminuria, smoking, alcohol consumption), and other examination factors (leg fat parameters other than main predictor, arm fat parameters other than main predictor). Lean/fat ratio and fat % variables were not included in the same model due to significant correlation and high variance inflation factor

When stratifying by BMI category, we found that BMI only modestly, insignificantly modified the effect of appendicular adiposity on BP (Tables S6, 7). In males, while leg/total% displayed a significant, inverse association with DBP across all BMI categories, the associations for leg lean/fat ratio and leg fat% were only significant in overweight and obese males (Table S6). In females, BMI stratification nullified the associations between appendicular adiposity and BP; only arm lean/fat ratio remained positively associated with SBP across all BMI categories (Table S7).

When we assessed continuous BP changes across tertiles of adiposity, males and females in the highest tertile (T3) of leg/total% had decreased mean DBP and those in T3 of leg lean/fat ratio had increased mean SBP, similar to findings treating adiposity as continuous (Table S3). As differences between males and females were not significant, initial analyses done combining sexes but still accounting for sex-specific tertiles were justified.

The predictive value of leg/total% was determined by estimating the AUC using the c-statistic. We found that leg/total% had a c-statistic of 0.644, whereas arm/total% had a c-statistic of 0.509. Leg/total% had similar, but slightly lower, c-statistic than both age and waist circumference and higher c-statistic than BMI (Table S10).

## Discussion

In this nationally representative study of 7411 young- to middle-aged adults, we found that leg adiposity, as measured via leg/total% and leg fat%, as well as corresponding arm adiposity measures were inversely associated with DBP and SBP, respectively. Arm and leg lean/fat ratio, on the other hand, were positively associated with SBP. Among the three measures of leg adiposity, only leg/total% demonstrated a significant, 30% decreased odds of hypertension when comparing T3 (≥36% for male, ≥41% for female) to T1 (< 31.5% for male, < 36% for female). Furthermore, leg/total% decreased the odds of IDH and SDH, but not ISH relative to normotension. BMI modified the association between leg/total% and hypertension, with low/normal BMI individuals showing significantly more protective odds than overweight and obese individuals. Interestingly, arm fat%, not arm/total%, was inversely associated with SBP and overall hypertension. Consequently, leg/total% had a higher predictive value than arm/total% but was insignificantly lower than waist circumference and age in predicting hypertension.

Our findings are in line with existing literature on the association between leg adiposity and various cardiometabolic conditions [13–32]. While prior studies have generally not focused on BP or hypertension as a primary outcome, they have consistently reported inverse associations between leg adiposity measures and metabolic risk factors. Zhang et al. [19], in a study of NHANES 1999 to 2006 participants with DXA scans, found that, regardless of ethnicity, leg adiposity indices (leg/total%, leg fat%, leg fat/truncal fat ratio) were all inversely associated with metabolic syndrome. Leg/total%, however, had the strongest association. Other studies have similarly found inverse associations between leg/total% and cardiovascular risk factors [18–20], cardiovascular disease risk and all-cause mortality [21–26]. In fact, Han et al. [25], using the Korean NHANES, showed that leg/total% displayed the strongest association with atherosclerotic cardiovascular disease risk. Interestingly, studies that additionally assessed arm adiposity have found no significant association, and in some cases positive associations, with metabolic risk factors [17, 20, 32, 33]. Our findings also corroborate a positive association between arm/total% and SBP and hypertension that was later attenuated by adjustment for cardiometabolic risk factors.

There continues to be variability in data concerning sex differences. Hu et al. [20], among White and Black Americans, showed that females generally had more protective associations of leg adiposity with metabolic syndrome criteria than males, Sakai et al. [18] demonstrated no difference between males and females for SBP or DBP after adjusting for menopausal status. While we also found no significant difference between sexes, we observed more protective odds of hypertension in females than males.

Of the four studies that have investigated the association between appendicular adiposity and BP/hypertension [18, 19, 25, 31], only one was conducted in a US representative population, all used 140/90 as the hypertension threshold, and none have commented on hypertension subtype. Nevertheless, our findings corroborate those of the four studies. Sakai et al. [18] found a significant, inverse association between leg fat mass and hypertension (≥140/90) as well as a mean 0.22 mmHg decrease in DBP and 0.16 mmHg decrease in SBP per 1 kg increase in leg fat mass in 4256 Japanese men aged 20 to 79 years. The authors also found an increase in BP for every 1 kg increase in lean leg mass, a counterintuitive finding that we also observed [18]. Increased lean mass, we speculate, may be associated with increased serum testosterone, an androgen that has been inconclusively linked to increased BP and cardiovascular disease [37]. Zhang et al. [19] showed a significant inverse correlation between leg/total%, but not leg fat/leg mass ratio, and SBP and DBP. Han et al. [25], in the Korean NHANES population, found that those in T1 of leg/total% had nearly 3.5 times the odds of hypertension (≥140/90) compared to T3, although models were not adjusted for truncal adiposity or arm adiposity measures. Finally, Yan et al. [31], in a Chinese population, similarly demonstrated that leg/total% was primary negative risk factor for hypertension (≥140/90).

They clarified that this association was stronger in the non-obese population, a finding that we also demonstrated. This would seem to suggest that, even in those with low/normal BMI, body distribution of fat is important. However, after adjusting for truncal fat and waist circumference, leg/total% continues to be a negative predictor of hypertension in low/normal BMI individuals. This suggests that, in addition to body distribution of fat, there exist a physiological difference between lower extremity adiposity and truncal or upper extremity adiposity. Conventionally, we have treated truncal fat as the primary source of fat that drives of metabolic disease; however, these studies and ours lead to the speculation that measurement of leg fat is just as important, and its effects independent of, truncal fat.

Not only is leg/total% inversely associated with hypertension, it is also preferentially inversely associated with DBP and associated hypertension subtypes. This suggests that leg adiposity may a play a role in regulation of DBP, which is determined in large part by peripheral vascular resistance in arteriolar vessels. Although the biological mechanism behind leg fat’s protective role is unknown, several studies have speculated and found that (1) leg fat is located primarily in subcutaneous tissue, as opposed to visceral tissue; (2) adipocytes in the lower extremities had lower free fatty acid turnover and lower rates of lipolysis; and (3) this decreased free fatty acid turnover may lead to decreased fatty acid concentrations in the blood and downregulation of triglyceride production [18, 19, 38].

We hypothesize that, in addition to decreased sympathetic and renin-angiotensin-aldosterone axis activation associated with subcutaneous fat, this downregulation of triglyceride production, which is corroborated by inverse associations between leg/total% and triglyceride levels in studies assessing metabolic syndrome criteria, leads to decreased endothelial cell damage in arterioles and maintenance of vessel elasticity and compliance. This is further supported by Lee et al. [24], which found that every SD increase in leg/total% was associated with a 1.03 m/sec decrease in brachial-ankle pulse wave velocity, a measure of arterial stiffness.

There are several strengths of this study. We used a set of recent, large, nationally representative surveys with gold standard measurements of fat and lean mass through DXA scans. We comprehensively accounted for covariates to further confirm the independent association of leg/total% and BP. By having a younger study population, we also minimized unmeasured chronic disease effects and effects of menopause on women. We utilized the 2017 ACC/AHA guidelines for hypertension, which not only includes more individuals in its hypertension diagnosis but emphasizes the notion that leg/total% may be important in those who are seemingly healthy.

Despite these strengths, there are limitations as well. This is a cross-sectional study so causality cannot be determined. It may be that those who are hypertensive tend to accumulate fat elsewhere. Further, while it is generalizable to those in the US aged 20 to 59 years, these results may not be generalizable to older adults ≥60 years who also have disproportionately higher hypertension prevalence. While DXA scans are the gold standard measurement for body fat distribution, it cannot distinguish between subcutaneous and intramuscular fat in the legs, limiting our interpretation of results. Furthermore, visceral fat is still more clinically relevant and more strongly associated with cardiovascular disease risk, posing the question as to how clinically useful our results may be. Also, although our results are statistically significant, they may or may not be clinically significant. Although the absolute changes in BP are miniscule, it is important to note these BP differences are on a population level and across 1% increments of appendicular adiposity. There may be residual confounding that is unaccounted for in this analysis, especially due to limited information on medication use and comorbidities (e.g., polycystic ovarian syndrome in women, hormone therapy).

## Conclusions

In conclusion, a greater proportional distribution of total body fat around the legs is inversely, independently associated with DBP, while a greater proportion of fat in one’s leg mass is inversely associated with SBP. Prospective studies on body fat distribution and incident hypertension are needed to understand the predictive value of leg adiposity and associated clinical surrogates (e.g., thigh circumference). These measures may help clinicians risk stratify patients to help improve hypertension screening and management. Technological improvements allowing for body part-specific fat measurement may also be in the horizon over the next decade and allow for further testing and clinical applicability of these leg adiposity measures.

## Supplementary Information

**Additional file 1: Table S1.** Baseline characteristics in females by hypertension status. **Table S2.** Baseline characteristics in males by hypertension status. **Table S3.** Differences in mean blood pressure by tertiles of leg adiposity measures. **Table S4.** Average appendicular adiposity by hypertension subtype. **Table S5.** Association between arm adiposity and blood pressure. **Table S6.** Association between appendicular adiposity and SBP by BMI group in males. **Table S7.** Association between appendicular adiposity and SBP by BMI group in females. **Table S8.** Association between continuous leg adiposity measures and hypertension subtypes. **Table S9.** Association between continuous arm adiposity measures and hypertension subtypes. **Table S10.** estimated AUC for traditional and novel risk factors. **Fig. S1.** Exclusion cascade for final study sample from National Health and Nutrition Examination Survey 2011–2018. **Fig. S2.** Correlation between leg adiposity measures and blood pressure. **Fig. S3.** Prevalence of hypertension subtypes by leg/total % tertiles.

## Data Availability

Data is available here: https://wwwn.cdc.gov/nchs/nhanes/continuousnhanes/overview.aspx?BeginYear=2011.

## Abbreviations

ACC/AHA: American College of Cardiology/American Heart Association
ALT: Alanine aminotransferase
AST: Aspartate aminotransferase
AUC: Area-under-receiver operator curve
BMI: Body mass index
BP: Blood pressure
CI: Confidence interval
DBP: Diastolic blood pressure
DXA: Dual-energy X-ray absorptiometry
HbA1c: Hemoglobin A1c
HDL: High density lipoprotein
HOMA-IR: Homeostatic Model Assessment of Insulin Resistance
HTN: Hypertension
IDH: Isolated diastolic hypertension
ISH: Isolated systolic hypertension
LDL: Low-density lipoprotein
NHANES: National Health and Nutrition Examination Survey
OR: Odds ratio
RRR: Relative risk ratio
SBP: Systolic blood pressure
SD: Standard deviation
SDH: Systolic-diastolic hypertension
SE: Standard error
β: Regression parameter estimate
