# Supplementary material for: Leg and arm adiposity is inversely associated with diastolic hypertension in young and middle-aged United States adults
Source: Clin Hypertens. 2022 Jan 15;28:3. doi: 10.1186/s40885-021-00190-2 (PMC8760692; doi:10.1186/s40885-021-00190-2)
Supplement: Supplementary file 1 — Additional file 1: Table S1. Baseline characteristics in females by hypertension status. Table S2. Baseline characteristics in males by hypertension status. Table S3. Differences in mean blood pressure by tertiles of leg adiposity measures. Table S4. Average appendicular adiposity by hypertension subtype. Table S5. Association between arm adiposity and blood pressure. Table S6. Association between appendicular adiposity and SBP by BMI group in males. Table S7. Association between appendicular adiposity and SBP by BMI group in females. Table S8. Association between continuous leg adiposity measures and hypertension subtypes. Table S9. Association between continuous arm adiposity measures and hypertension subtypes. Table S10. estimated AUC for traditional and novel risk factors. Fig. S1. Exclusion cascade for final study sample from National Health and Nutrition Examination Survey 2011–2018. Fig. S2. Correlation between leg adiposity measures and blood pressure. Fig. S3. Prevalence of hypertension subtypes by leg/total % tertiles. [file 40885_2021_190_MOESM1_ESM.docx]

**Table S1.** Baseline characteristics in females by hypertension status

| Variable | Overall  3,688 (100) | Hypertension^a)^  695 (17.8%) | Normotension  2,993 (82.2%) | Overall^b)^  P-value |
| --- | --- | --- | --- | --- |
| **Demographics** |  |  |  |  |
| Age (yr), mean (SD) | 37.8 (0.3) | 43.9 (0.6) | 36.5 (0.3) | <0.0001 |
| Race/ethnicity |  |  |  |  |
| Mexican American | 603 (10.5%) | 103 (9.2%) | 500 (10.7%) | 0.0015 |
| Other Hispanic | 416 (7.5%) | 73 (6.3%) | 343 (7.8%) |  |
| Non-Hispanic White | 1,335 (63.1%) | 221 (60.0%) | 1,114 (63.8%) |  |
| Non-Hispanic Black | 639 (9.3%) | 157 (12.8%) | 482 (8.5%) |  |
| Other race | 695 (9.6%) | 141 (11.6%) | 554 (9.2%) |  |
| Poverty-income ratio | 2.99 (0.06) | 3.08 (0.09) | 2.97 (0.06) | 0.2659 |
| Education level |  |  |  |  |
| <High school | 553 (10.3%) | 111 (11.1%) | 442 (10.1%) | 0.3045 |
| High school | 688 (17.6%) | 155 (19.5%) | 533 (17.2%) |  |
| >High school | 2,447 (72.1%) | 429 (69.4%) | 2,018 (72.7%) |  |
| **Sociobehavioral factors** |  |  |  |  |
| Smoking status^c)^ |  |  |  |  |
| Low/none | 1,317 (39.1%) | 258 (42.7%) | 1,059 (38.4%) | 0.1653 |
| Moderate | 1,603 (39.8%) | 285 (35.3%) | 1,318 (40.8%) |  |
| High | 768 (21.0%) | 152 (22.0%) | 616 (20.8%) |  |
| Alcohol consumption (drinks/day) | 2.5 (0.2) | 2.4 (0.1) | 2.5 (0.2) | 0.5543 |
| **Examination measurements** |  |  |  |  |
| BMI (kg/m^2^) | 27.9 (0.2) | 30.5 (0.4) | 27.3(0.2) | <0.0001 |
| Waist circumference (cm) | 93.0 (0.4) | 99.7 (0.9) | 91.6 (0.4) | <0.0001 |
| Truncal fat percent (%) | 35.3 (0.2) | 38.3 (0.4) | 34.6 (0.3) | <0.0001 |
| % Total fat in lower extremities^d)^ | 39.2 (0.1) | 37.0 (0.3) | 39.7 (0.2) | <0.0001 |
| Leg lean mass/fat mass ratio | 1.33 (0.01) | 1.28 (0.02) | 1.34 (0.01) | 0.0011 |
| Leg fat % (% of total leg mass) | 42.6 (0.2) | 43.4 (0.3) | 42.4 (0.2) | 0.0012 |
| Average leg area (cm^2^) | 341.0 (0.8) | 344.5 (1.9) | 340.3 (0.9) | 0.0438 |
| Upper leg length (cm) | 37.9 (0.1) | 37.5 (0.2) | 38.0 (0.1) | 0.0109 |
| % Total fat in upper extremities | 12.2 (0.0) | 12.4 (0.1) | 12.2 (0.0) | 0.0149 |
| Arm lean mass/fat mass ratio | 1.4 (0.0) | 1.3 (0.0) | 1.5 (0.0) | <0.0001 |
| Arm fat % (% of total arm mass) | 41.4 (0.2) | 43.6 (0.3) | 41.0 (0.3) | <0.0001 |
| Average arm area (cm^2^) | 209.0 (0.6) | 214.2 (1.5) | 207.9 (0.6) | 0.0002 |
| Heart rate (bpm) | 74.2 (0.3) | 75.9 (0.7) | 73.9 (0.3) | 0.0090 |
| SBP (mmHg) | 113.3 (0.3) | 130.0 (0.7) | 109.7 (0.2) | <0.0001 |
| DBP (mmHg) | 69.2 (0.3) | 80.0 (0.4) | 66.9 (0.3) | <0.0001 |
| **Laboratory measurements** |  |  |  |  |
| Plasma glucose (mg/dL) | 97.8 (0.6) | 104.8 (1.7) | 96.5 (0.7) | <0.0001 |
| Hemoglobin A1c (%) | 5.38 (0.01) | 5.59 (0.04) | 5.33 (0.02) | <0.0001 |
| Serum insulin (uU/mL) | 10.7 (0.2) | 13.8 (1.0) | 10.2 (0.3) | 0.0010 |
| Serum HDL (mg/dL) | 59.2 (0.5) | 58.3 (1.2) | 59.4 (0.6) | 0.4170 |
| Serum LDL (mg/dL) | 111.9 (1.5) | 121.1 (5.3) | 110.4 (1.6) | 0.0628 |
| Serum triglycerides (mg/dL) | 117.2 (2.0) | 144.1 (5.1) | 111.4 (2.0) | <0.0001 |
| ALT (U/L) | 20.1 (0.3) | 23.1 (0.8) | 19.5 (0.3) | 0.0002 |
| AST (U/L) | 22.1 (0.2) | 24.1 (0.9) | 21.6 (0.2) | 0.0074 |
| Serum albumin (g/dL) | 4.23 (0.01) | 4.19 (0.02) | 4.23 (0.01) | 0.0136 |
| Serum creatinine | 0.730 (0.004) | 0.728 (0.008) | 0.731 (0.005) | 0.6870 |
| Serum creatine phosphokinase | 105.9 (2.8) | 105.2 (3.5) | 106.0 (3.0) | 0.8142 |
| **Comorbidities** |  |  |  |  |
| Impaired glucose tolerance^e)^ | 1,028 (27.8%) | 290 (41.7%) | 738 (24.6%) | <0.0001 |
| Hypertriglyceridemia | 888 (22.9%) | 247 (36.3%) | 641 (20.0%) | <0.0001 |
| Anti-diabetes medication use | 104 (1.9%) | 23 (2.9%) | 81 (1.7%) | 0.061 |
| Cholesterol medication use | 123 (3.7%) | 31 (5.0%) | 92 (3.4%) | 0.13 |
| HOMA-IR^f)^ | 415 (28.3%) | 105 (46.9%) | 310 (24.8%) | <0.0001 |

Continuous variables represented as weighted mean (SD). Categorical variables represented as unweighted N (weighted %). SD, standard deviation; BMI, body mass index; SBP, systolic blood pressure; DBP, diastolic blood pressure; HDL, high density lipoprotein; LDL, low density lipoprotein; ALT, alanine aminotransferase; AST, aspartate aminotransferase; HOMA-IR, Homeostatic Model Assessment of Insulin Resistance.

^a)^Hypertension defined as SBP ≥130 mmHg or DBP ≥80 mmHg or self-reported hypertension diagnosis. ^b)^P-value calculated using one-way ANOVA for continuous variables and Rao-Scott chi square tests for categorical variables. P-values and/or significance levels were not adjusted for multiple comparisons as this table was considered exploratory in nature. ^c)^Smoking categories are based off serum cotinine levels which represent exposure to nicotine (low: <0.015 ng/ml, medium: 0.015-3 ng/ml, high: >3 ng/ml). ^d)^% Total fat in lower extremities corresponds to leg/total %, leg lean mass/fat mass ratio corresponds to leg lean/fat ratio, % fat in total leg mass corresponds to leg fat %. Identical for arm measures. ^e)^Impaired glucose tolerance was defined as hemoglobin A1c ≥5.7%, fasting plasma glucose ≥ 100 mg/dL, self-reported history of diabetes or self-reported history of oral anti-diabetes medications or insulin therapy. ^f)^Data on HOMA-IR only available for 1,359 females. HOMA = fasting glucose (mg/dL) × insulin (uU/mL) / 405. HOMA ≥3 signifies insulin resistance.

**Table S2.** Baseline characteristics in males by hypertension status

| **Variable** | **Overall**  3,723 (100%) | **Hypertension**^a)^  1,158 (29.7%) | **Normotension**  2,565 (70.3%) | P-value ^b)^ |
| --- | --- | --- | --- | --- |
| **Demographics** |  |  |  |  |
| Age (yr) | 36.9 (0.3) | 40.1 (0.6) | 35.5 (0.3) | <0.0001 |
| Race/ethnicity |  |  |  |  |
| Mexican American | 597 (12.3%) | 167 (11.2%) | 430 (12.8%) | 0.054 |
| Other Hispanic | 372 (7.6%) | 114 (7.6%) | 258 (7.6%) |  |
| Non-Hispanic White | 1,293 (60.4%) | 387 (59.6%) | 906 (60.8%) |  |
| Non-Hispanic Black | 690 (9.7%) | 257 (11.8%) | 433 (8.9%) |  |
| Other race | 771 (10.0%) | 233 (9.9%) | 538 (10.0%) |  |
| Poverty-income ratio | 2.99 (0.07) | 3.00 (0.08) | 2.98 (0.07) | 0.7572 |
| Education level |  |  |  |  |
| <High school | 2,111 (62.4%) | 641 (61.2%) | 1,470 (62.9%) | 0.4964 |
| High school | 885 (23.6%) | 281 (25.1%) | 604 (23.0%) |  |
| >High school | 727 (14.0%) | 236 (13.7%) | 491 (14.1%) |  |
| **Sociobehavioral factors** |  |  |  |  |
| Smoking status^c)^ |  |  |  |  |
| Low/none | 930 (29.5%) | 289 (29.5%) | 641 (29.5%) | 0.6430 |
| Moderate | 1,448 (36.7%) | 443 (35.4%) | 1,005 (37.3%) |  |
| High | 1,341 (33.8%) | 426 (35.2%) | 915 (33.2%) |  |
| Alcohol consumption (drinks/day) | 3.8 (0.3) | 3.6 (0.1) | 4.0 (0.4) | 0.3015 |
| **Examination measurements** |  |  |  |  |
| BMI (kg/m^2^) | 27.9 (0.1) | 29.7 (0.2) | 27.1 (0.2) | <0.0001 |
| Waist circumference (cm) | 97.3 (0.4) | 102.3 (0.6) | 95.2 (0.4) | 0.0001 |
| Truncal fat percent (%) | 26.7 (0.2) | 28.8 (0.2) | 25.8 (0.2) | <0.0001 |
| % Total fat in lower extremities^d)^ | 34.0 (0.1) | 32.6 (0.2) | 34.6 (0.2) | <0.0001 |
| Leg lean mass/fat mass ratio | 2.71 (0.02) | 2.61 (0.04) | 2.75 (0.03) | 0.0029 |
| Leg fat % (% of total leg mass) | 27.4 (0.2) | 28.1 (0.3) | 27.1 (0.2) | 0.0031 |
| Average leg area (cm^2^) | 402.4 (1.0) | 404.8 (1.7) | 401.3 (1.0) | 0.0364 |
| Upper leg length (cm) | 41.7 (0.1) | 41.7 (0.1) | 41.6 (0.1) | 0.8275 |
| % Total fat in upper extremities | 11.77 (0.03) | 11.76 (0.05) | 11.77 (0.03) | 0.8560 |
| Arm lean mass/fat mass ratio | 3.20 (0.02) | 2.98(0.04) | 3.30 (0.03) | <0.0001 |
| Arm fat % (% of total arm mass) | 24.5 (0.1) | 25.8 (0.2) | 23.9 (0.2) | <0.0001 |
| Average arm area (cm^2^) | 255.1 (0.7) | 259.5 (1.2) | 253.3 (0.7) | <0.0001 |
| Heart rate (bpm) | 71.2 (0.3) | 74.4 (0.5) | 69.9 (0.3) | <0.0001 |
| Systolic blood pressure (mmHg) | 119.6 (0.3) | 131.0 (0.5) | 114.7 (0.2) | <0.0001 |
| Diastolic blood pressure (mmHg) | 72.1 (0.3) | 81.2 (0.5) | 68.2 (0.3) | <0.0001 |
| **Laboratory measurements** |  |  |  |  |
| Plasma glucose (mg/dL) | 102.6 (0.7) | 107.6 (1.32) | 100.8 (0.8) | <0.0001 |
| Hemoglobin A1c (%) | 5.43 (0.02) | 5.54 (0.03) | 5.39 (0.02) | <0.0001 |
| Serum insulin (uU/mL) | 11.9 (0.5) | 14.0 (0.8) | 11.2 (0.6) | 0.0044 |
| Serum HDL (mg/dL) | 48.2 (0.3) | 48.0 (0.6) | 48.3 (0.4) | 0.6934 |
| Serum LDL (mg/dL) | 116.9 (1.0) | 121.8 (3.0) | 115.3 (1.3) | 0.0912 |
| Serum triglycerides (mg/dL) | 163.0 (3.2) | 194.4 (5.1) | 150.0 (3.4) | <0.0001 |
| ALT (U/L) | 29.8 (0.4) | 34.1 (0.9) | 27.9 (0.6) | <0.0001 |
| AST (U/L) | 26.6 (0.3) | 28.8 (0.6) | 25.6 (0.4) | 0.0003 |
| Serum albumin (g/dL) | 4.46 (0.01) | 4.45 (0.01) | 4.46 (0.01) | 0.2282 |
| Serum creatinine | 0.958 (0.004) | 0.958 (0.007) | 0.958 (0.004) | 0.9696 |
| Serum creatine phosphokinase | 206.7 (5.6) | 202.6 (6.9) | 208.4 (7.7) | 0.5939 |
| **Comorbidities** |  |  |  |  |
| Impaired glucose tolerance^e)^ | 1,377(31.0%) | 521(41.3%) | 856(19.6%) | <0.0001 |
| Hypertriglyceridemia | 1,504 (40.2%) | 597 (52.3%) | 907 (35.1%) | <0.0001 |
| Anti-diabetes medication use | 86 (1.5%) | 30 (1.6%) | 56 (1.5%) | 0.87 |
| Cholesterol medication use | 141 (4.5%) | 54 (5.9%) | 87 (4.0%) | 0.069 |
| HOMA-IR^f)^ | 474 (33.5%) | 191 (46.6%) | 283 (28.8%) | <0.0001 |

Continuous variables represented as weighted mean (SD). Categorical variables represented as unweighted N (weighted %).

SD, standard deviation; BMI, body mass index; HDL, high density lipoprotein; LDL, low density lipoprotein; ALT, alanine aminotransferase; AST, aspartate aminotransferase; HOMA-IR, Homeostatic Model Assessment of Insulin Resistance.

^a)^Hypertension defined as SBP ≥130 mmHg or DBP ≥80 mmHg or self-reported hypertension diagnosis. ^b)^P-value calculated using one-way ANOVA for continuous variables and Rao-Scott chi square tests for categorical variables. P-values and/or significance levels were not adjusted for multiple comparisons as this table was considered exploratory in nature. ^c)^Smoking categories are based off serum cotinine levels which represent exposure to nicotine (low: <0.015 ng/ml, medium: 0.015-3 ng/ml, high: >3 ng/ml). ^d)^% Total fat in lower extremities corresponds to leg/total %, leg lean mass/fat mass ratio corresponds to leg lean/fat ratio, % fat in total leg mass corresponds to leg fat %. Identical for arm measures. ^e)^Impaired glucose tolerance was defined as hemoglobin A1c ≥5.7%, fasting plasma glucose ≥ 100 mg/dL, self-reported history of diabetes or self-reported history of oral anti-diabetes medications or insulin therapy. ^f)^Data on HOMA-IR only available for 1,397 males. HOMA = fasting glucose (mg/dL) × insulin (uU/mL) / 405. HOMA ≥3 signifies insulin resistance.

**Table S3.** Differences in blood pressure by tertiles of leg adiposity measures

| Variable | | | SBP | | DBP | |
| --- | --- | --- | --- | --- | --- | --- |
|  |  |  | β (95% CI) | P-value | β (95% CI) | P-value |
| No. (%) | 1,853 (23.8) | - | - | - | - | - |
| Leg/total % | Male | Tertile 1 | REF |  | REF |  |
|  |  | Tertile 2 | –1.52 (–2.88, –0.15) | 0.031 | –1.76 (–2.87, –0.65) | 0.0024 |
|  |  | Tertile 3 | –1.47 (–2.98, 0.04) | 0.056 | –3.27 (–4.53, –2.01) | <0.0001 |
|  | Female | Tertile 1 | REF |  | REF |  |
|  |  | Tertile 2 | –1.89 (–3.31, –0.48) | 0.010 | –1.24 (–2.35, –0.12) | 0.03 |
|  |  | Tertile 3 | –2.29 (–4.13, –0.44) | 0.016 | –1.67 (–2.89, –0.45) | 0.0079 |
| Leg lean/fat ratio | Male | Tertile 1 | REF |  | REF |  |
|  |  | Tertile 2 | 1.68 (0.38, 2.99) | 0.012 | –0.14 (–1.26, 0.97) | 0.81 |
|  |  | Tertile 3 | 1.44 (–0.25, 3.13) | 0.094 | –1.13 (–2.80, 0.54) | 0.18 |
|  | Female | Tertile 1 | REF |  | REF |  |
|  |  | Tertile 2 | 0.72 (–0.73, 2.17) | 0.74 | 0.33 (–0.63, 1.29) | 0.50 |
|  |  | Tertile 3 | 1.85 (0.22, 3.48) | 0.027 | 0.65 (–0.69, 2.00) | 0.34 |
| Leg fat % | Male | Tertile 1 | REF |  | REF |  |
|  |  | Tertile 2 | 0.14 (–1.33, 1.62) | 0.84 | 0.75 (–0.45, 1.95) | 0.22 |
|  |  | Tertile 3 | –1.35 (–3.16, 0.45) | 0.14 | 1.34 (–0.37, 3.04) | 0.12 |
|  | Female | Tertile 1 | REF |  | REF |  |
|  |  | Tertile 2 | –1.25 (–2.63, 0.14) | 0.078 | –0.48 (–1.40, 0.43) | 0.30 |
|  |  | Tertile 3 | –1.35 (–2.85, 0.14) | 0.076 | –0.66 (–2.01, 0.69) | 0.34 |

SBP, systolic blood pressure; DBP, diastolic blood pressure; β, regression parameter estimate; CI, confidence interval (All estimates are of the fully adjusted model.).

Model 1 adjusts for demographic factors (age, sex, race/ethnicity, poverty index). Model 2 adjusts for Model 1 Factors + cardiometabolic factors (SBP, heart rate, triglycerides, body mass index, truncal fat mass, high density lipoprotein, alanine aminotransferase, diabetes status, serum creatinine, albuminuria, smoking, alcohol consumption).

Model 3 adjusts Model 2 parameters + other examination factors (leg fat parameters other than main predictor, arm fat parameters other than main predictor).

Fully adjusted model is identical to Model 3. Model 3 adjusts for other examination factors (leg fat parameters other than main predictor, or arm fat parameters other than main predictor). Lean/fat ratio and fat % variables were not included in the same model due to significant correlation and high variance inflation factor. % Total fat in lower extremities corresponds to leg/total %, leg lean mass/fat mass ratio corresponds to leg lean/fat ratio, % fat in total leg mass corresponds to leg fat %. Identical for arm measures. Tertiles are as follows: leg/total % (female: <36%, 36%–41%, ≥41%; male: <31.5%, 31.5%–36.0%, ≥36%); leg lean/fat ratio (female: <1.1, 1.1–1.4, ≥1.40; male: <2.3, 2.3–3.0, ≥3.0); leg fat % (female: <40%, 40%–45%, ≥45%; male: <24%, 24%–29%, ≥29%).

**Table S4**. Average appendicular adiposity by hypertension subtype

| Variable | | Hypertension subtype | | | P for SDH  vs. IDH^a)^ | P for ISH  vs. IDH^a)^ |
| --- | --- | --- | --- | --- | --- | --- |
|  |  | IDH | SDH | ISH |  |  |
| Leg/total % | Unadjusted | 34.2 ± 0.2 | 33.3 ± 0.3 | 35.0 ± 0.3 | 0.0760 | 0.1142 |
|  | Fully adjusted^b)^ | 36.2 ± 0.2 | 36.0 ± 0.3 | 37.1 ± 0.3 | 0.5190 | 0.0311 |
| Arm/total % | Unadjusted | 11.89 ± 0.06 | 11.96 ± 0.09 | 12.15 ± 0.08 | 0.5019 | 0.0131 |
|  | Fully adjusted^b)^ | 12.01 ± 0.07 | 12.03 ± 0.08 | 12.25 ± 0.08 | 0.8205 | 0.0139 |
| Leg lean/fat ratio | Unadjusted | 2.11 ± 0.05 | 2.16 ± 0.06 | 2.13 ± 0.07 | 0.5806 | 0.8211 |
|  | Fully adjusted | 2.16 ± 0.04 | 2.24 ± 0.04 | 2.23 ± 0.04 | 0.0562 | 0.1930 |
| Arm lean/fat ratio | Unadjusted | 2.36 ± 0.06 | 2.35 ± 0.07 | 2.41 ± 0.07 | 0.98 | 0.60 |
|  | Fully adjusted | 2.38 ± 0.02 | 2.40 ± 0.02 | 2.49 ± 0.03 | 0.67 | 0.0071 |
| Leg fat % | Unadjusted | 33.5 ± 0.5 | 33.4 ± 0.6 | 34.1 ± 0.6 | 0.8993 | 0.4253 |
|  | Fully adjusted | 33.6 ± 0.3 | 33.0 ± 0.3 | 33.4 ± 0.4 | 0.0647 | 0.6009 |
| Arm fat % | Unadjusted | 31.9 ± 0.5 | 32.2 ± 0.6 | 32.5 ± 0.6 | 0.7221 | 0.4892 |
|  | Fully adjusted | 32.4 ± 0.2 | 32.4 ± 0.2 | 32.0 ± 0.2 | 0.9699 | 0.1423 |

Data are presented as mean ± standard error.

SDH, systolic-diastolic hypertension; IDH, isolated diastolic hypertension; ISH, isolated systolic hypertension.

^a)^P determined using fully adjusted model using one-way ANOVA with post-hoc Tukey’s pairwise comparison tests. ^b)^Fully adjusted model is identical to Model 3, excluding blood pressure. Model 1 adjusts for demographic factors (age, sex, race/ethnicity, poverty index). Model 2 adjusts for Model 1 Factors + cardiometabolic factors (SBP, heart rate, triglycerides, body mass index, truncal fat mass, high density lipoprotein, alanine aminotransferase, diabetes status, serum creatinine, albuminuria, smoking, alcohol consumption). Model 3 adjusts Model 2 parameters + other examination factors (leg fat parameters other than main predictor, arm fat parameters other than main predictor).

**Table S5. Association between arm adiposity and blood pressure**

| **Variable** | | | **SBP** | | **DBP** | | **Pulse pressure** | |
| --- | --- | --- | --- | --- | --- | --- | --- | --- |
|  |  |  | β (95% CI) | P-value | β (95% CI) | P-value | β (95% CI) | P-value |
| No. (%) | 1,853 (23.8) |  | - | - | - | - | - | - |
| Arm/total % | Male | Unadjusted | 0.044 (–0.37, 0.45) | 0.83 | –0.68 (–1.13, –0.23) | 0.0037 | 0.72 (0.33, 1.11) | 0.0005 |
|  |  | Fully adjusted^a)^ | –0.44 (–0.82, –0.067) | 0.021 | –0.82 (–1.22, –0.43) | 0.0001 | 0.38 (–0.033, 0.80) | 0.071 |
|  | Female | Unadjusted | 0.78 (0.42, 1.14) | <0.0001 | 0.072 (–0.22, 0.37) | 0.62 | 0.71 (0.42, 1.00) | <0.0001 |
|  |  | Fully adjusted | –0.030 (–0.40, 0.34) | 0.87 | –0.40 (–0.73, –0.067) | 0.019 | 0.37 (–0.001, 0.74) | 0.051 |
| Arm lean/fat ratio | Male | Unadjusted | –1.25 (–1.72, –0.79) | <0.0001 | –1.79 (–2.11, –1.45) | <0.0001 | 0.53 (0.052, 0.99) | 0.030 |
|  |  | Fully adjusted | 1.52 (0.40, 2.63) | 0.0086 | 0.90 (–0.15, 1.94) | 0.093 | 0.62 (–0.52, 1.76) | 0.28 |
|  | Female | Unadjusted | –3.41 (–4.24, –2.58) | <0.0001 | –2.19 (–2.88, –1.51) | <0.0001 | –1.22 (–2.11, –0.31) | 0.0090 |
|  |  | Fully adjusted | 2.08 (0.47, 3.68) | 0.012 | 0.62 (–0.86, 2.11) | 0.41 | 1.46 (–0.44, 3.36) | 0.13 |
| Arm fat% | Male | Unadjusted | 0.24 (0.15, 0.33) | <0.0001 | 0.27 (0.21, 0.33) | <0.0001 | –0.032 (–0.11, 0.051) | 0.44 |
|  |  | Fully adjusted | –0.26 (–0.43, –0.098) | 0.0023 | –0.21 (–0.35, –0.067) | 0.0046 | –0.054 (–0.21, 0.10) | 0.49 |
|  | Female | Unadjusted | 0.26 (0.20, 0.32) | <0.0001 | 0.15 (0.099, 0.19) | <0.0001 | 0.11 (0.048, 0.18) | 0.0009 |
|  |  | Fully adjusted | –0.22 (–0.33, –0.10) | 0.0004 | –0.12 (–0.24, 0.0049) | 0.060 | –0.099 (–0.26, 0.057) | 0.21 |

SBP, systolic blood pressure; DBP, diastolic blood pressure; β, regression parameter estimate; CI, confidence interval (All estimates are of the fully adjusted model.).

^a)^Fully adjusted model is identical to Model 3 and adjusts for demographic factors (age, sex, race/ethnicity, poverty index), cardiometabolic factors (SBP, heart rate, triglycerides, body mass index, truncal fat mass, high density lipoprotein, alanine aminotransferase, diabetes status, serum creatinine, albuminuria, smoking, alcohol consumption), and other examination factors (leg fat parameters other than main predictor, arm fat parameters other than main predictor)**.** Lean/fat ratio and fat % variables were not included in the same model due to significant correlation and high variance inflation factor.

**Table S6. Association between appendicular adiposity and SBP by BMI group in males**

| **Variable** | | **BMI group** | | |
| --- | --- | --- | --- | --- |
|  |  | Low/normal | Overweight | Obese |
| Leg/total% | DBP | –0.32 (–0.55, –0.10)^**^ | –0.23 (–0.44, –0.03)^*^ | –0.43 (–0.72, –0.15)^**^ |
|  | SBP | –0.22 (–0.45, 0.03) | –0.02 (–0.26, 0.23) | –0.14 (–0.44, 0.16) |
| Arm/total% | DBP | –0.07 (–0.94, 0.80) | –0.57 (–1.21, 0.07) | –0.97 (–1.56, –0.38)^**^ |
|  | SBP | –0.77 (–1.48, –0.07)^*^ | 0.66 (–0.04, 1.36) | 0.23 (–0.43, 0.88) |
| Leg lean/fat ratio | DBP | –0.65 (–1.66, 0.36) | 0.38 (–1.28, 2.05) | –2.13 (–4.46, 0.20) |
|  | SBP | –0.22 (–1.21, 0.78) | 2.86 (1.42, 4.30) ^***^ | 2.06 (–0.26, 4.38) |
| Arm lean/fat ratio | DBP | –0.22 (–1.05, 0.30) | 0.85 (–0.54, 2.25) | –0.05 (–1.85, 1.76) |
|  | SBP | 0.21 (–0.62, 1.05) | 2.93 (1.85, 4.00)^***^ | 3.04 (1.04, 5.04)^**^ |
| Leg fat% | DBP | 0.16 (–0.06, 0.38) | –0.08 (–0.29, 0.14) | 0.11 (–0.18, 0.41) |
|  | SBP | –0.07 (–0.10, 0.24) | –0.39 (–0.62, –0.16)^**^ | –0.29 (–0.55, –0.04)^*^ |
| Arm fat% | DBP | 0.08 (–0.17, 0.32) | –0.22 (–0.45, –0.002) ^*^ | –0.04 (–0.27, 0.20) |
|  | SBP | –0.12 (–0.43, 0.19) | –0.20 (–0.52, 0.13) | –0.31 (–0.56, –0.07)^*^ |

Data are presented as regression parameter estimate (95% confidence interval).

SBP, systolic blood pressure; BMI, body mass index; DBP, diastolic blood pressure (All estimates are of the fully adjusted model.).

^*^P < 0.05; ^**^ P < 0.01; ^***^ P < 0.001.

Fully adjusted model is identical to Model 3 and adjusts for demographic factors (age, sex, race/ethnicity, poverty index), cardiometabolic factors (systolic BP, heart rate, triglycerides, body mass index, truncal fat mass, high density lipoprotein, alanine aminotransferase, diabetes status, serum creatinine, albuminuria, smoking, alcohol consumption), and other examination factors (leg fat parameters other than main predictor, arm fat parameters other than main predictor). Lean/fat ratio and fat % variables were not included in the same model due to significant correlation.

**Table S7. Association between appendicular adiposity and SBP by BMI group in females**

| **Variable** | | **BMI group** | | |
| --- | --- | --- | --- | --- |
|  |  | Low/normal | Overweight | Obese |
| No. (% HTN) |  | 443 (16.9) | 612 (31.7) | 798 (52.1) |
| Leg/total % | DBP | –0.15 (–0.31, 0.004) | –0.18 (–0.44, 0.07) | –0.003 (–0.23, 0.23) |
|  | SBP | –0.18 (–0.39, 0.03) | –0.16 (–0.44, 0.13) | –0.10 (–0.37, 0.18) |
| Arm/total % | DBP | 0.11 (–0.34, 0.57) | –0.56 (–1.11, –0.01)^*^ | –0.11 (–0.64, 0.43) |
|  | SBP | 0.74 (0.01, 1.47)^*^ | 0.57 (–0.11, 1.26) | 0.58 (0.09, 1.07)^*^ |
| Leg lean/fat ratio | DBP | –0.08 (–1.86, 1.69) | 0.42 (–3.17, 4.00) | 1.28 (–2.76, 5.31) |
|  | SBP | 0.63 (–1.60, 2.86) | 3.55 (–0.45, 7.56) | 2.78 (–0.88, 6.44) |
| Arm lean/fat ratio | DBP | 0.27 (–0.96, 1.50) | 0.82 (–2.47, 4.11) | 1.40 (–2.43, 5.23) |
|  | SBP | 1.64 (0.15, 3.13)^*^ | 6.09 (2.78, 9.40)^***^ | 6.22 (3.05, 9.38)^***^ |
| Leg fat % | DBP | –0.03 (–0.15, 0.10) | –0.04 (–0.25, 0.17) | –0.08 (–0.30, 0.14) |
|  | SBP | –0.05 (–0.21, 0.11) | –0.20 (–0.45, 0.05) | –0.16 (–0.36, 0.03) |
| Arm fat % | DBP | –0.05 (–0.17, 0.07) | –0.07 (–0.28, 0.13) | –0.10 (–0.27, 0.07) |
|  | SBP | –0.01 (–0.21, 0.19) | –0.23 (–0.51, 0.05) | –0.20 (–0.41, –0.04)^*^ |

Data are presented as regression parameter estimate (95% confidence interval).

SBP, systolic blood pressure; BMI, body mass index; HTN, hypertension; DBP, diastolic blood pressure (All estimates are of the fully adjusted model.).

^*^P < 0.05; ^***^P < 0.001.

Fully adjusted model is identical to Model 3 and adjusts for demographic factors (age, sex, race/ethnicity, poverty index), cardiometabolic factors (SBP, heart rate, triglycerides, body mass index, truncal fat mass, high density lipoprotein, alanine aminotransferase, diabetes status, serum creatinine, albuminuria, smoking, alcohol consumption), and other examination factors (leg fat parameters other than main predictor, arm fat parameters other than main predictor). Lean/fat ratio and fat % variables were not included in the same model due to significant correlation.

**Table S8.** Association between continuous leg adiposity measures and hypertension subtypes

| Variable | | Hypertension Subtype | Hypertension overall |
| --- | --- | --- | --- |
|  |  | RRR (95% CI) | OR (95% CI) |
| Leg/total % | Normotension | 1.00 (ref) |  |
|  | IDH | 0.96 (0.93, 0.98) | 0.97 (0.95, 0.99) |
|  | SDH | 0.94 (0.91, 0.97) |  |
|  | ISH | 1.01 (0.97, 1.04) |  |
| Leg lean/fat ratio | Normotension | 1.00 (ref) |  |
|  | IDH | 0.80 (0.62, 1.03) | 1.11 (0.95, 1.29) |
|  | SDH | 1.17 (0.82, 1.66) |  |
|  | ISH | 1.13 (0.81, 1.58) |  |
| Leg fat % | Normotension | 1.00 (ref) |  |
|  | IDH | 1.00 (0.95, 1.05) | 1.00 (0.97, 1.04) |
|  | SDH | 0.96 (0.90, 1.02) |  |
|  | ISH | 1.02 (0.94, 1.09) |  |

RRR, relative risk ratio; CI, confidence interval; OR, odds ratio; IDH, isolated diastolic hypertension; SDH, systolic-diastolic hypertension; ISH, isolated systolic hypertension.

All estimates shown are fully adjusted via model 3. Model 3 adjusts for other examination factors (leg fat parameters other than main predictor, arm fat parameters other than main predictor). % Total fat in lower extremities corresponds to leg/total %, leg lean mass/fat mass ratio corresponds to leg lean/fat ratio, % fat in total leg mass corresponds to leg fat %. Identical for arm measures. Tertiles are as follows: leg/total % (female: <36%, 36%–41%, ≥41%; male: <31.5%, 31.5%–36.0%, ≥36%); leg lean/fat ratio (female: <1.1, 1.1–1.4, ≥1.4; male: <2.3, 2.3–3.0, ≥3.0); leg fat % (female: <40%, 40%–45%, ≥45%; male: <24%, 24%–29%, ≥29%).

**Table S9.** Association between continuous arm adiposity measures and hypertension subtypes

| Variable | | Hypertension Subtype | Hypertension overall |
| --- | --- | --- | --- |
|  |  | RRR (95% CI) | OR (95% CI) |
| Arm/total % | Normotension | 1.00 (ref) |  |
|  | IDH | 0.90 (0.82, 0.99) | 0.98 (0.92, 1.06) |
|  | SDH | 0.95 (0.86, 1.05) |  |
|  | ISH | 1.05 (0.95, 1.17) |  |
| Arm lean/fat ratio | Normotension | 1.00 (ref) |  |
|  | IDH | 0.95 (0.79, 1.13) | 1.13 (1.00, 1.27) |
|  | SDH | 1.19 (0.98, 1.45) |  |
|  | ISH | 0.89 (0.66, 1.22) |  |
| Arm fat % | Normotension | 1.00 (ref) |  |
|  | IDH | 1.00 (0.97, 1.02) | 0.98 (0.96, 0.99) |
|  | SDH | 0.97 (0.95, 1.00) |  |
|  | ISH | 0.95 (0.92, 0.98) |  |

RRR, relative risk ratio; CI, confidence interval; OR, odds ratio; IDH, isolated diastolic hypertension; SDH, systolic-diastolic hypertension; ISH, isolated systolic hypertension.

All estimates shown are fully adjusted via model 3. Model 3 adjusts for other examination factors (leg fat parameters other than main predictor, arm fat parameters other than main predictor). % Total fat in upper extremities corresponds to arm/total %, arm lean mass/fat mass ratio corresponds to arm lean/fat ratio, % fat in total arm mass corresponds to arm fat %. Tertiles are as follows: arm/total % (female: <11.6%, 11.6%–12.9%, ≥12.9%; male: <11.3%, 11.3%–12.2%, ≥12.2%); arm lean/fat ratio (female: <1.10, 1.10–1.50, ≥1.50; male: <2.60, 2.60–3.50, ≥3.50); arm fat % (female: <39%, 39%–45%, ≥45%; male: <21%, 21%–26%, ≥26%).

**Table S10.** Estimated AUC for traditional and novel risk factors

| **Variable** | **Estimated AUC for hypertension** | **Estimated AUC for hypertension (treating variable as continuous)** |
| --- | --- | --- |
| **Traditional risk factors** |  |  |
| Age | 0.630 | 0.645 |
| Smoking | 0.520 | 0.522 |
| BMI | 0.607 | 0.621 |
| Waist circumference | 0.575 | 0.646 |
| **Novel factors** |  |  |
| Leg/total % | 0.600 | 0.644 |
| Arm/total % | 0.525 | 0.509 |

AUC, area-under-receiver operator curve; BMI, body mass index.


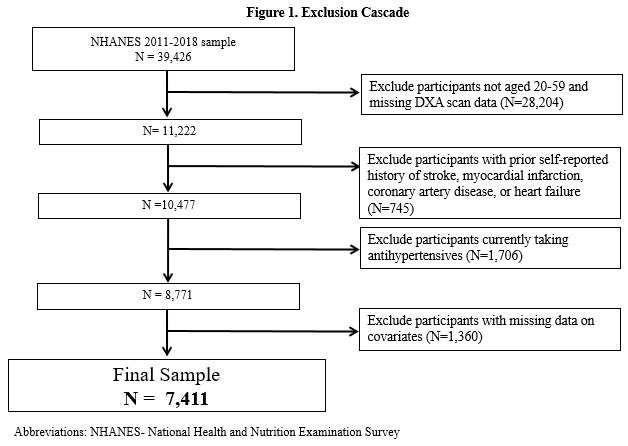
**Fig. S1.** exclusion cascade for final study sample from National Health and Nutrition Examination Survey 2011–2018.

**Fig. S2. Correlation between leg adiposity measures and blood pressure.** Scatter plot depicts a representative 20% subsample. Circles correspond to individual participants. Lines represent best fit lines via least mean squares estimates. Correlation coefficients and corresponding p-values are shown in the bottom right corner. Red, female; Black, male; SBP, systolic blood pressure; DBP, diastolic blood pressure.


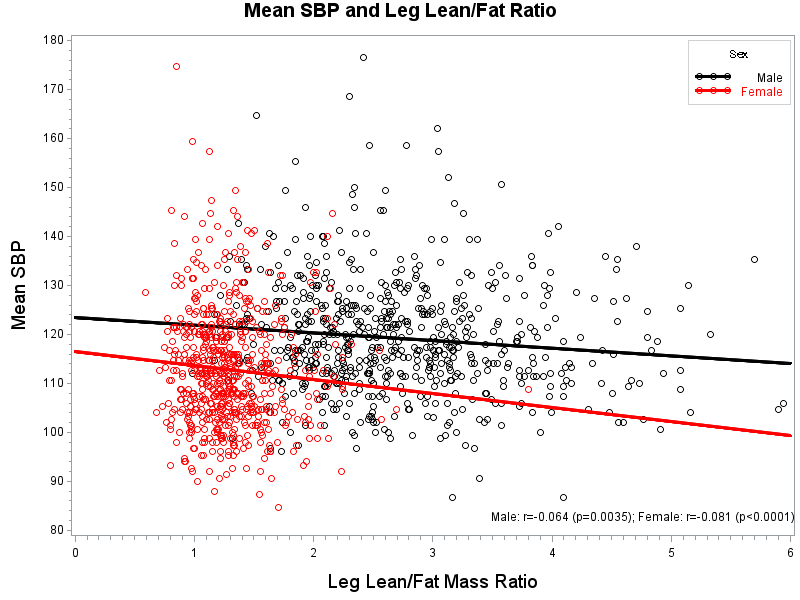

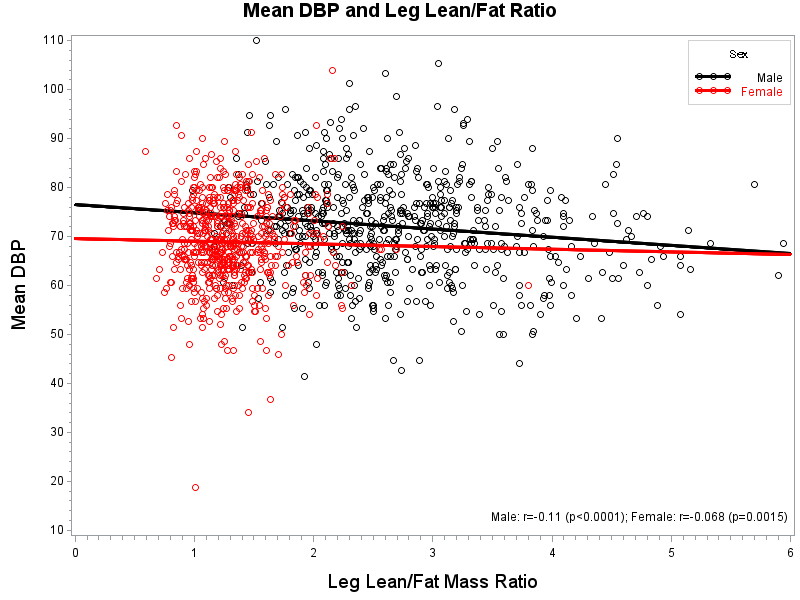

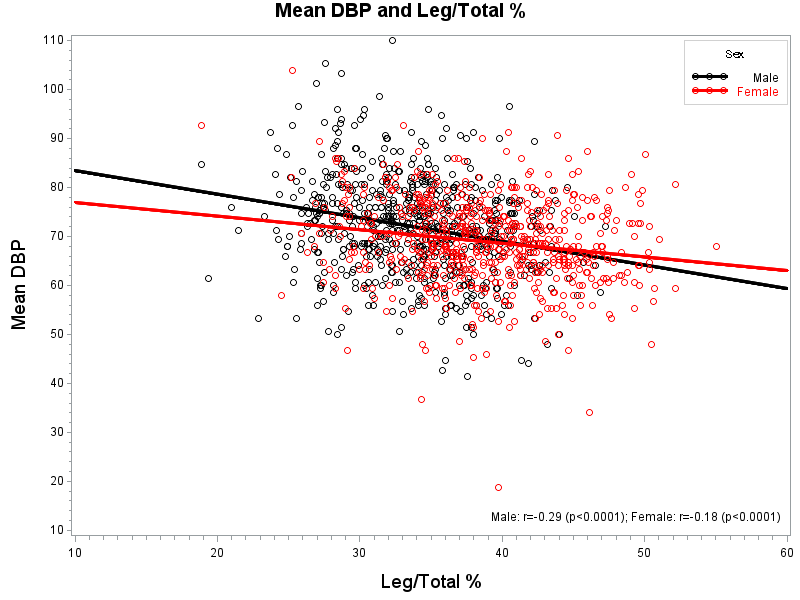

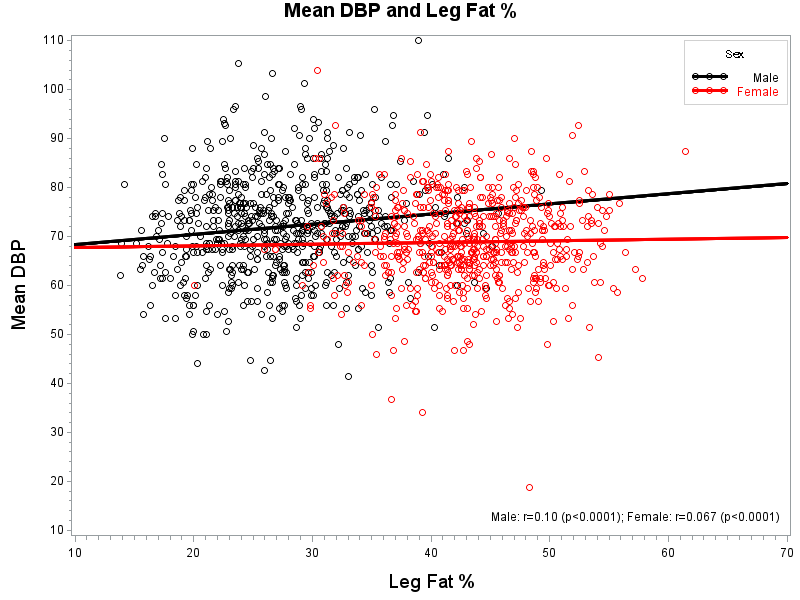

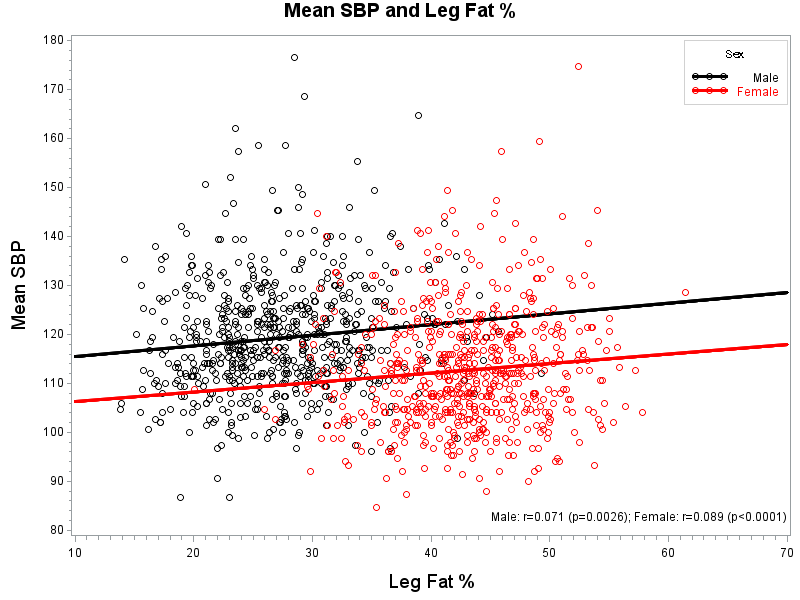

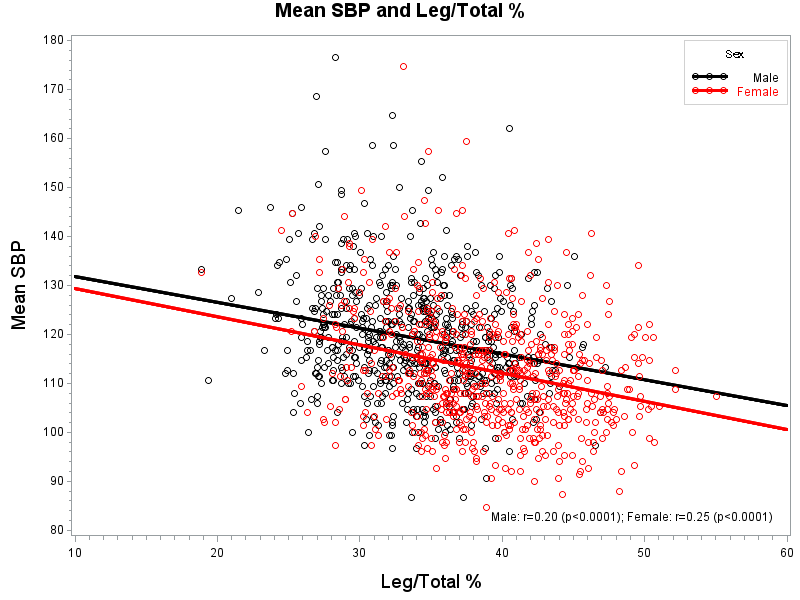


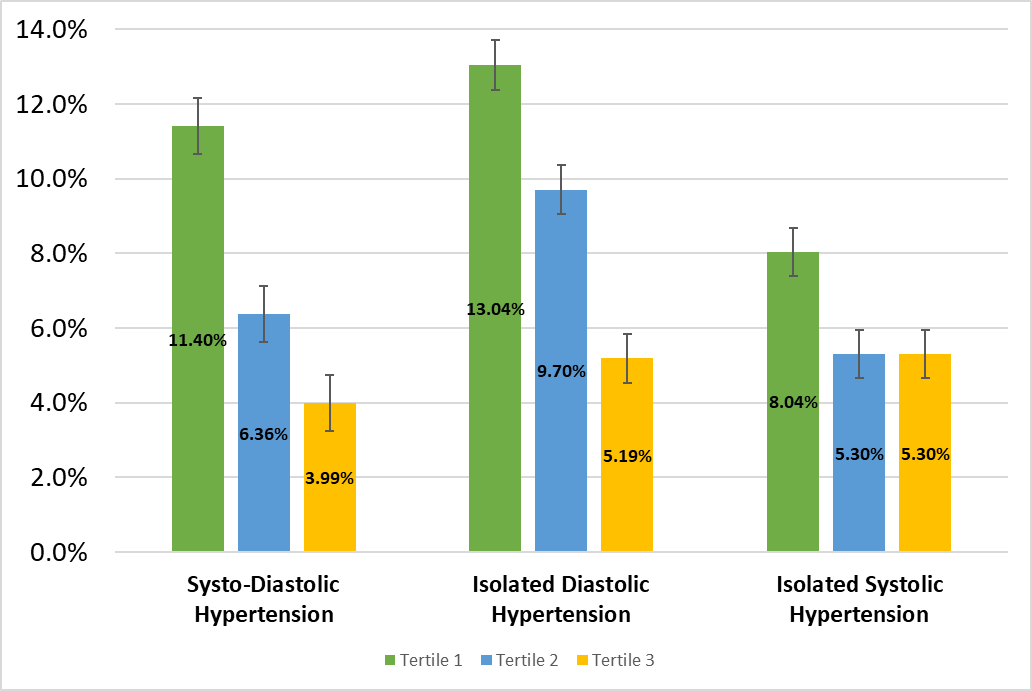


**Fig. S3. Prevalence of hypertension subtypes by leg/total % tertiles.** Green bars represent the lowest tertile. Blue bars represent the middle tertile. Orange bars represent the highest tertile. Proportion of hypertension subtype within each tertile is shown, with the standard deviation represented by error bars.
